# Intestinal organoids model human responses to infection by commensal and Shiga toxin producing *Escherichia coli*

**DOI:** 10.1371/journal.pone.0178966

**Published:** 2017-06-14

**Authors:** Sayali S. Karve, Suman Pradhan, Doyle V. Ward, Alison A. Weiss

**Affiliations:** 1 Department of Molecular Genetics, Biochemistry, and Microbiology, University of Cincinnati, Cincinnati, Ohio, United States of America; 2 Center for Microbiome Research and Department of Microbiology and Physiological Systems, University of Massachusetts Medical School, Worcester, Massachusetts, United States of America; New York State Department of Health, UNITED STATES

## Abstract

Infection with Shiga toxin (Stx) producing *Escherichia coli* O157:H7 can cause the potentially fatal complication hemolytic uremic syndrome, and currently only supportive therapy is available. Lack of suitable animal models has hindered study of this disease. Induced human intestinal organoids (iHIOs), generated by in vitro differentiation of pluripotent stem cells, represent differentiated human intestinal tissue. We show that iHIOs with addition of human neutrophils can model *E*. *coli* intestinal infection and innate cellular responses. Commensal and O157:H7 introduced into the iHIO lumen replicated rapidly achieving high numbers. Commensal *E*. *coli* did not cause damage, and were completely contained within the lumen, suggesting defenses, such as mucus production, can constrain non-pathogenic strains. Some O157:H7 initially co-localized with cellular actin. Loss of actin and epithelial integrity was observed after 4 hours. O157:H7 grew as filaments, consistent with activation of the bacterial SOS stress response. SOS is induced by reactive oxygen species (ROS), and O157:H7 infection increased ROS production. Transcriptional profiling (RNAseq) demonstrated that both commensal and O157:H7 upregulated genes associated with gastrointestinal maturation, while infection with O157:H7 upregulated inflammatory responses, including interleukin 8 (IL-8). IL-8 is associated with neutrophil recruitment, and infection with O157:H7 resulted in recruitment of human neutrophils into the iHIO tissue.

## Introduction

Shiga toxin producing *E*. *coli* (STEC), including O157:H7 are an important cause of diarrheal disease, causing about 265,000 illnesses yearly in the US [1]. Shiga toxin (Stx) is responsible for the life-threatening systemic complication, hemolytic uremic syndrome (HUS). Currently, supportive therapy is the only treatment, and importantly, patients treated with antibiotics are more are more likely to develop severe disease, including HUS [2]. Stx is an AB_5_ toxin; the B-pentamer promotes entry of A-subunit into the mammalian cytoplasm, and the enzymatic A-subunit damages ribosomes, inhibiting protein synthesis [3]. Recent studies have shown that the A- and B-subunits can circulate independently, and active toxin is formed by subunit association on the target cell surface [4]. The genes for Stx are encoded in the late-gene region of lysogenic bacteriophages, and are silent until viral lytic replication is triggered by the bacterial SOS stress response [5]. Some antibiotics upregulate Stx expression, and this is likely responsible for the association of antibiotic treatment with increased risk for severe disease [6–8].

O157:H7 do not naturally infect mice, and there is a need for human model systems. Remarkable progress has been made using human tissue specific stem-cell propagated enteroids [9]. Especially important has been the demonstration that the previously noncultivatable pathogen, human norovirus, can replicate in human intestinal enteroids [10]. Enteroids have been used to model bacterial infection, including studies with *E*. *coli* O157:H7 [11]. Some studies have been performed using enteroid monolayers in transwells, with tissue culture medium on both the apical and basolateral surfaces. The presence of glucose-rich medium on the apical surface does not replicate the nutrient environment in the intestinal lumen, and presents a technical problem for studying bacteria, such as *E*. *coli*, which replicate rapidly and produce bi-products that can be toxic to cells. Furthermore, the smaller size of enteroids makes microinjection challenging.

**Fig 1:**
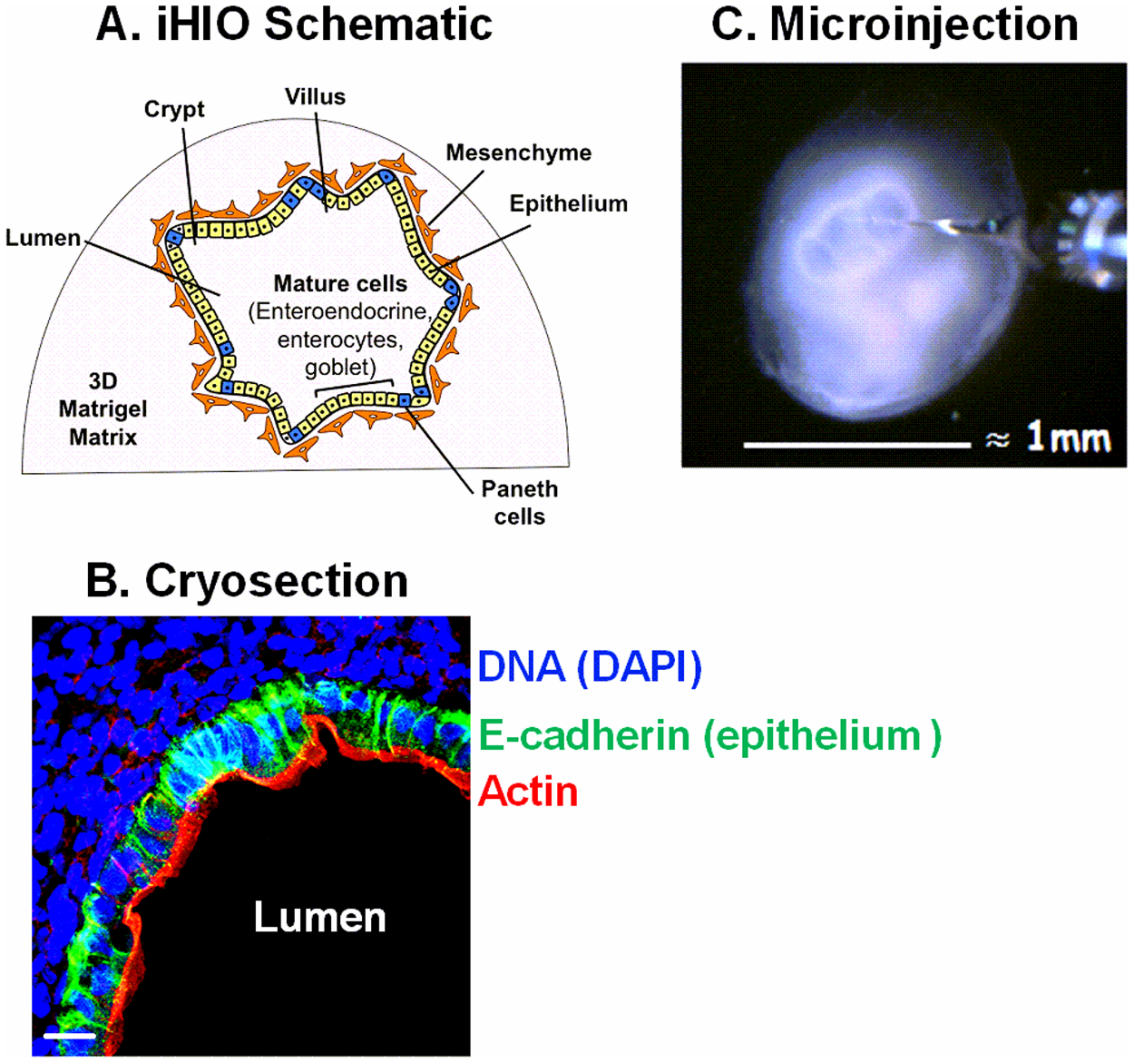
Components of iHIOs. (A) Diagram of cross section of iHIO embedded in Matrigel (extracellular matrix). (B) Histologic staining of iHIO 24 hours after microinjection with PBS; merged image stained for DNA (DAPI, blue) and antibodies to the epithelial marker E-cadherin (green), and F-actin (red). Confocal images were captured using Zeiss LSM710 Live Duo confocal microscope and merged using ImageJ software. Bar indicates 20 μm. (C) Picture of an iHIO in the process of microinjection.

Pluripotent stem-cell “induced human intestinal organoids” (iHIOs) represent a new experimental model to study enteric pathogens [12,13]. iHIOs are generated entirely in vitro from pluripotent embryonic stem cells by a process that mimics normal differentiation [12], and represent a potentially infinite source of identical tissue samples. iHIOs represent human tissue from the distal portion of the small intestine [12], the tissues favored for initial attachment of *E*. *coli* O157:H7 [14]. iHIOs adopt the three-dimensional architecture of the human intestine (Fig 1) and contain a luminal cavity bounded by a single epithelial layer, and resemble sterile neonatal tissue [15]. Microscopic brush borders, and villus and crypt structures are formed [12]. The epithelium contains absorptive enterocytes and the major secretory lineages (paneth cells, enteroendocrine cells, and goblet cells), and intestinal functions such as peptide transport and mucus secretion by goblet cells are maintained [12]. The epithelium is surrounded by a stratified mesenchyme which contains smooth muscle cells and sub-epithelial fibroblasts [12]. iHIOs have been successfully used to model features of embryonic development [16] and inflammatory bowel disease [17]. In this study, iHIOs were infected with commensal as well as pathogenic *E*. *coli* O157:H7. The iHIOs were not damaged by infection with commensal *E*. *coli*; however, O157:H7 produced a very severe and rapid loss of epithelial structural integrity.

## Materials and methods

### *E*. *coli* strains

We used strains characterized in previous studies. Bacterial assemblies and sequence data for strains ECOR13 and PT29 are deposited under NCBI BioProject ID: PRJNA359210. *E*. *coli* O157:H7, PT29S, is a spontaneous streptomycin resistant mutant of PT29S previously isolated from a patient [18]. PT29 is sequence type ST-11 (https://cge.cbs.dtu.dk/services/MLST/) and only possesses the genes for the more potent from of Shiga toxin, Shiga toxin type 2 (Stx2), not Shiga toxin type 1 (Stx1). In addition to Stx, the Virulence Finder search program (https://cge.cbs.dtu.dk/services/VirulenceFinder/), revealed PT29 possessed virulence factor genes typical for O157:H7 including; *tir* (translocated intimin receptor protein), *eae* (intimin), *espA* (type III secretion system), *espB* (secreted protein B), *espF* (type III secretion system), *ehxA* (enterohaemolysin), *espP* (extracellular serine protease plasmid-encoded), *iss* (increased serum survival), *espJ* (prophage-encoded type III secretion system effector), *etpD* (type II secretion protein), *astA* (EAST-1 heat-stable toxin), *nleA-C* (non-LEE encoded effectors A-C), *katP* (plasmid-encoded catalase peroxidase), *toxB* (toxin B), and *iha* (adherence protein).

Commensal strain SGUC183, also known as 183ϕS [8] is streptomycin and gentamicin resistant derivative of ECOR13, a non-pathogenic *E*. *coli* isolated from a healthy person in Sweden, and is part of the Michigan State University STEC Center ECOR collection [8]. ECOR13 is Group A, Sequence Type ST-44. The only hit using the Virulence Finder program was glutamate decarboxylase (GadB), which converts glutamate to gamma-aminobutyrate. This activity helps to maintain neutral intracellular pH following exposure to extremely acidic conditions, such as transit through the stomach [19]. Streptomycin resistance was selected as a spontaneous mutation; gentamicin resistance was introduced by transformation with plasmid pBBR1MCS-5 [20].

### Reagents and equipment used in iHIO culture

Matrigel basement membrane matrix (BD Biosciences, cat. 356234), and extracellular matrix gel (Sigma, cat. E1270) were used to embed the iHIOs in order to support development of 3-dimensional architecture. Gut media for iHIO culture was prepared using advanced Dulbecco's Modified Eagle Medium/Ham's F-12 (DMEM/F12) (Gibco, Invitrogen, cat. 12634–028) supplemented with B27 insulin (Invitrogen, cat. 17504044), N2 supplement (Invitrogen, cat. 17502048), 2 mM L-glutamine (Fisher, cat. SH3003401), 15 mM HEPES (Invitrogen, cat. 15630080), 100 ng ml^-1^epidermal growth factor (R&D Systems, cat. 236-EG-200), and either 2 mM penicillin/streptomycin (Invitrogen, cat. 15140–122) or penicillin alone (Amresco, cat. E480-20ML). iHIOs were maintained in tissue culture treated Nucleon delta 4-well (Nunc, cat. 176740) and 2-well (Lab-Tek, cat. 155380) dishes.

iHIOs prepared by directed differentiation of H1 human embryonic stem cell line (NIH registry number 0043) [12] were obtained from Pluripotent Stem Cell Facility and Organoid Core at Cincinnati Children’s Hospital and Medical Center. iHIOs were maintained in reconstituted gut media, which was changed twice weekly.

#### Microinjection of iHIOs

Drummond glass capillaries (Fisher, cat. 21-171-4) were pulled with a micropipette puller (Sutter Instrument Company). The sealed tips of the capillaries were cut open using Cuterz glass scissors, and the capillaries were loaded onto Nanoject II auto-nanoliter injector (Fisher, cat. 13-681-455). Microinjections were performed, and before and after injection images of iHIOs were obtained using a stereomicroscope (Leica). In some studies iHIOs were co-injected with 2.5 mg ml^-1^ of fluorescent dye fluorescein isothiocyanate (FITC) to label the lumen and to assess maintenance of the epithelial barrier as reported in previous studies [21]. The iHIOs were incubated at 37°C in a humidified chamber containing 5% CO_2_ for 5 days. For bacterial infections, approximately 10^3^ *E*. *coli* cells were microinjected into the iHIO lumen. The iHIOs infected with *E*. *coli* were incubated in reconstituted gut media containing penicillin (final concentration 100 U ml^-1^) at 37°C in a humidified chamber with 5% CO_2_ for 1 day. Images of the injected iHIOs were collected using Zeiss LSM710 Live Duo Confocal Microscope.

#### Cryosectioning and staining of iHIOs

iHIOs were fixed in 4% paraformaldehyde (2 to 4 hours) followed by 30% sucrose (overnight). Organoids were prepared for cryosectioning by freezing at -20°C in Tissue Freezing Medium (Fisher, cat. 15-183-13). Cryosections (10 μm) were prepared with BD Cryotome FSE Cryostat and the sections placed on a plus glass microscope slides. Histologic stains are listed in S1 Table. To stain, sections were fixed in cold acetone (10 minutes), rinsed with distilled water, and blocked in blocking buffer (PBS containing 10% goat serum, 1% bovine serum albumin (BSA) and 0.01% Triton X-100) for 2 hours at room temperature in a humidified chamber. The slides were drained and stained with primary antibody (1:500) in blocking buffer in a humidified chamber at 4°C overnight. Sections were rinsed twice with wash buffer (PBS with 0.1% BSA and 0.025% Triton X-100), and secondary antibody was diluted in PBS (1:1000) and was applied to the sections. The sections were allowed to incubate with the secondary antibody for 2 hours in dark at room temperature. The sections were washed with PBS and DNA was counterstained with Hoechst (1 μg/mL) or DAPI (0.5 μg/mL) dye for 2 minutes in dark. Stained sections were air-dried and mounted using VectaMount permanent mounting medium. Analysis was performed using Zeiss LSM710 Live Duo Confocal Microscope. Merged images were generated and the FITC fluorescence was quantified by using ImageJ software.

### Growth of *E*. *coli* in organoids

For challenge studies, approximately 10^3^ *E*. *coli* cells were microinjected into the iHIO lumen. After indicated incubation times at 37°C with 5% CO_2_ in a humidified chamber, the organoids were removed from the 3-dimensional culture matrix, transferred to an eppendorf tube, and washed with ice cold PBS. The organoids were then transferred to a sterile 2-ml tissue homogenizer, disrupted, and suspended in 100 μl PBS. Subsequent dilutions were plated on L-agar plates, and incubated at 37°C overnight. The total number of bacteria per organoid was calculated based on the colony forming units (CFU) observed on the agar plates on the next day.

Antibiotics are needed to confine bacterial growth to the lumen. Except where indicated, bacterial challenge studies were performed with streptomycin-resistant strains using extracellular matrix without gentamicin, and tissue culture media supplemented with penicillin and streptomycin. Penicillin-sensitive strains were able to grow in the organoid lumen, but not in the tissue culture medium when penicillin was in the tissue culture media. Gentamicin and streptomycin inhibited growth of antibiotic sensitive *E*. *coli* C600 strain [7] injected into the lumen. Matrigel is not available without antibiotics, so we transferred the organoids to the antibiotic-free extracellular matrix gel (Sigma Aldrich, cat. # E1270) when working with gentamicin-sensitive strains.

### Western blots to quantify Stx production

The iHIOs were infected with 10^3^ PT29S cells and incubated at 37°C with 5% CO_2_ in a humidified chamber. After the indicated incubation times, the iHIO suspension was obtained as described above. No signal was detected in the supernatants of the lysed organoids (data not shown). To determine whether the toxin was bound to the cell membrane, the lysate was centrifuged at 8600 x g 5 minutes at 4°C and the supernatant and pellet fractions were analyzed by Western blot.

Proteins were resolved in Bio-Rad Mini PROTEAN Tetra Cell using the 4–15% precast Mini-PROTEAN TGX^™^ gel. Samples were boiled for 7 minutes in sample buffer (1M Tris, pH 6.8, 50% glycerol, 10% SDS, 0.5% bromophenol blue, 0.5% beta-mercaptoethanol) before being loaded in a 15 μL volume. Gels were run at a constant 30 milliamps until the bromophenol blue dye reached the bottom of the gel. Proteins were transferred to a PVDF membrane in a Hoefer TE series transphor electrophoresis unit at 100 V for 1 hour using chilled transfer buffer (10% methanol, 24 mM Tris pH 8.3, 194 mM glycine). After transfer, the PVDF membrane was wetted in 100% methanol for 1 minute followed by PBS for two minutes. The membrane was incubated with primary antibody rabbit polyclonal recognizing Stx2 A- and B-subunits (1:5000) in Odyssey blocking diluent with 2% Tween 20, overnight followed by three washes in PBS-T (PBS with 0.1% Tween 20). IRDye 800CW Diluted Secondary antibody (Goat anti-rabbit) (1:10,000) in Odyssey blocking diluent with 0.2% Tween 20 was added to the membrane and incubated in the dark for one hour at room temperature with gentle shaking. The membrane was rinsed with PBS-T with vigorous shaking for 5 minutes. The washing was repeated three times and finally rinsed with PBS to remove the residual Tween 20 before the membrane was imaged in the Odyssey^®^ Family Imaging System (LI-COR; Odyssey CLx Near-Infrared (NIR) imaging system) for the presence of Stx2a. Purified Stx2a at 25 and 50 ng was used as the positive control. The respective protein bands were quantified compared to the Stx2a standards using LI-COR Image Studio 4.0 software.

### Assessment of production of reactive oxygen species

iHIOs were infected with 10^3^ commensal or 10^3^ pathogenic O157:H7 in a medium devoid of antibiotics and incubated at 37°C with 5% CO_2_ in a humidified chamber for a period of 4 h. Saline alone injected organoids were used as controls. At the indicated time, the iHIOs were again injected with 230 nL at 830 nM concentration of ROS detection reagent from Enzo Life Sciences. The iHIOs were further incubated at 37°C with 5% CO_2_ in a humidified chamber for an hour and the fluorescent intensity observed under a fluorescent microscope (Nikon Eclipse TE2000-U) and the fluorescence quantitated by image processing program ImageJ.

### Isolation and labeling of PMNs

De-identified human peripheral blood was obtained from the Cell Processing Core at Cincinnati Children’s Hospital Medical Center. 5.0 ml of blood in EDTA was carefully layered onto 5.0 ml of Polymorphprep^™^ (Axis-Shield, Cat # 2017–11), and centrifuged at 500 G for 35 min at room temperature. The upper band of plasma and mononuclear cells was removed, and the lower band of PMNs was harvested. An equal volume of half-strength HEPES-buffered saline (0.425% (w/v) NaCl, 5 mM HEPES-NaOH, pH 7.4) was added to the PMN suspension. The PMNs were harvested by centrifugation at 400 G for 10 min at room temperature and suspended in the modified gut medium. Cell counts were performed using the 40μm Scepter^™^ Cell Counter Sensor (Millipore, Cat # PHCC40050). The purified PMNs were labeled with 5 μM CellTracker^™^ Violet BMQC dye (Cat # C10094, Molecular Probes) for 30 minutes, centrifuged to remove excess dye and the washed PMNs were suspended at the required number in gut medium.

### Quantification of PMN migration into the iHIO tissues

iHIOs were injected with approximately 10^3^ *E*. *coli* or vehicle and incubated at 37°C with 5% CO_2_ in a humidified chamber. For experiments without antibiotics, after injection the organoids were washed 3 times with sterile PBS to remove extracellular bacteria. After 4 hours, 5 X 10^4^ PMNs in 20 μL were added to the wells and incubated for the indicated times. Fluorescent intensity of the labeled PMNs was observed on intact organoids by confocal microscope (Zeiss LSM710 LIVE Duo), and quantified by image processing using ImageJ. A standard plane of focus was used for all confocal images; the presence of the green FITC fluorescence indicates the image included the luminal compartment. The outline of the bright field image was used to define the boundaries of the organoid, and violet fluorescence within the boundary was considered to be due to internalized PMNs. Values were normalized to account for difference in the organoid image size. Bacterial numbers were determined as indicated above.

### Bioinformatics RNA-seq data analysis

RNA-seq was performed by Genomics, Epigenomics and Sequencing Core (GESC) in the University of Cincinnati. For each treatment, the total RNA from three independent iHIOs was extracted by using mirVana miRNA Isolation Kit (Lifetech, Grand Island, NY) with total RNA extraction protocol. Briefly, iHIOs were lyzed with lysis/binding buffer, treated with homogenate additive, and extracted with acid-phenol:chloroform. The supernatant was mixed with ethanol and passed through the filter cartridge. Bound RNA was washed and eluted. RNA concentrations were determined by Nanodrop (Thermo Scientific, Wilmington, DE), and integrity was determined by Bioanalyzer (Agilent, Santa Clara, CA). The Apollo 324 system (WaferGen, Fremont, CA) and PrepX PolyA script was used for automatic polyA RNA isolation. The library was prepared using PrepX mRNA Library kit (WaferGen) and Apollo 324 NGS automatic library prep system. Isolated RNA was RNase III fragmented, adaptor-ligated and converted to cDNA with Superscript III reverse transcriptase (Lifetech, Grand Island, NY), followed by automatic purification using Agencourt AMPure XP beads (Beckman Coulter, Indianapolis IN). The targeted cDNA fragments were around 200 base pairs (bp). Universal (SR) and index-specific primers were added to each adaptor-ligated cDNA sample and the amplified library was enriched by AMPure XP beads purification, and quality and yield of the library was assessed by Kapa Library Quantification kit (Kapabiosystem, Woburn, MA) using ABI's 9700HT real-time PCR system (Lifetech). Individually indexed libraries were proportionally pooled (20–50 million reads per sample) for clustering in cBot system (Illumina, San Diego, CA). Libraries at the final concentration of 15.0 pM were clustered onto a single read (SR) flow cell using Illumina’s TruSeq SR Cluster kit v3, and sequenced for 50 bp using TruSeq SBS kit on Illumina HiSeq system.

To analyze differential gene expression, sequence reads were aligned to the human genome using the TopHat aligner [22], and reads aligning to each known transcript were counted using Bioconductor packages for next-generation sequencing data analysis [23]. The differential expression analysis between different sample types was performed for each gene separately using the *edgeR* Bioconductor package [24]. The statistical significance of differential expression is established based on the FDR (False discovery rate)-adjusted p-values and are indicated as the values in the padj columns in S2 and S3 Tables) [25]. 19,076 transcripts were characterized; 18,543 were identified as genes and 15,448 were associated with a gene ontology (GO) term using the gene ontology analysis program GOrilla [26]. As expected transcripts (e.g. IL-13, IL-25, IL-22, INF-γ, TNF, and IL-12) restricted to hematopoietic lineages were not detected. Venn diagrams were prepared using the online tool from Bioinformatics & Evolutionary Genomics (http://bioinformatics.psb.ugent.be/webtools/Venn/).

## Results and discussion

### Sensitivity to LPS depends on route of exposure

**Fig 2:**
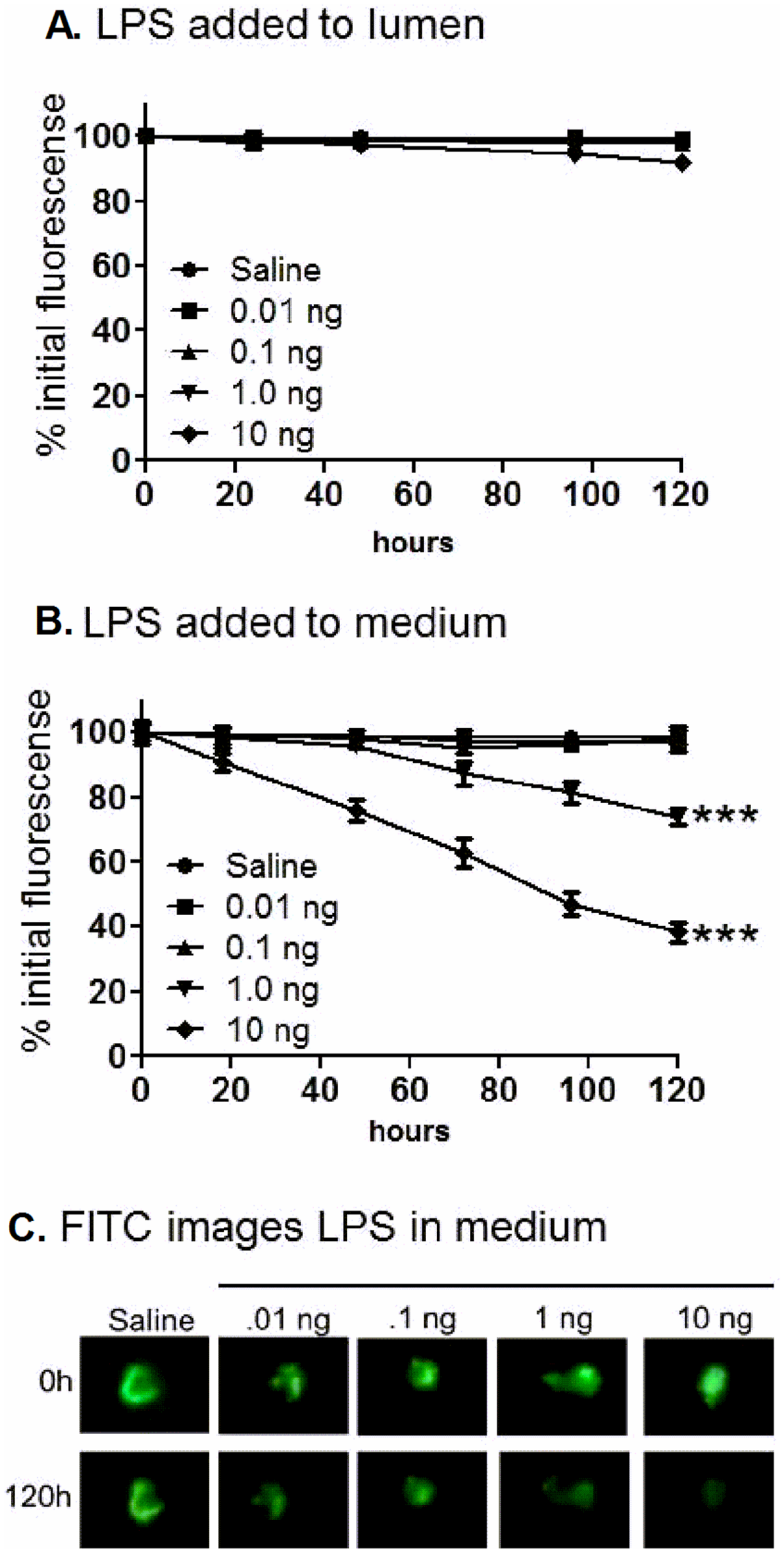
Sensitivity to LPS. Various doses of purified LPS were (A) microinjected into the iHIO lumen; or (B) introduced into the medium. Epithelial barrier function was monitored by quantifying retention of the fluorescent dye, FITC (C). Statistical significance of loss of fluorescence at 120 hours compared to control was assessed by GraphPad Prism 5 using one way ANOVA, Dunnett’s multiple comparison posttest. ***, extremely significant, P < 0.001, compared to saline control at 120 hours.

iHIOs resemble sterile neonatal tissue [15]. Microbial colonization promotes maturation of the neonatal intestine, and Gram negative lipopolysaccharide (LPS) elicits strong responses, which are dependent on the cell-surface that is exposed. For iHIOs, introduction of LPS into the lumen mimics natural intestinal colonization, while addition of LPS to the tissue culture medium mimics life-threatening septicemia. To assess LPS toxicity, the lumen was labeled with the fluorescent dye, fluorescein isothiocyanate (FITC), and the fluorescence was monitored to indicate maintenance of barrier function (Fig 2). Luminal addition of up to 10 ng of LPS did not compromise barrier function (Fig 2A). This intraluminal concentration is about 20,000 ng/ml, assuming a spherical organoid with a diameter of 1 mm has a volume of about 0.5 μL. In contrast, iHIOs were extremely sensitive to LPS added to the surrounding medium (Fig 2B and 2C); barrier function loss was seen when 1 ng was added to 0.5 ml media, for a concentration of 2 ng/ml. Thus, the luminal surface of iHIOs can tolerate high levels of LPS, but iHIOs are extremely sensitive to LPS introduced from the serosal side.

### iHIOs support luminal growth of *E*. *coli*

**Fig 3:**
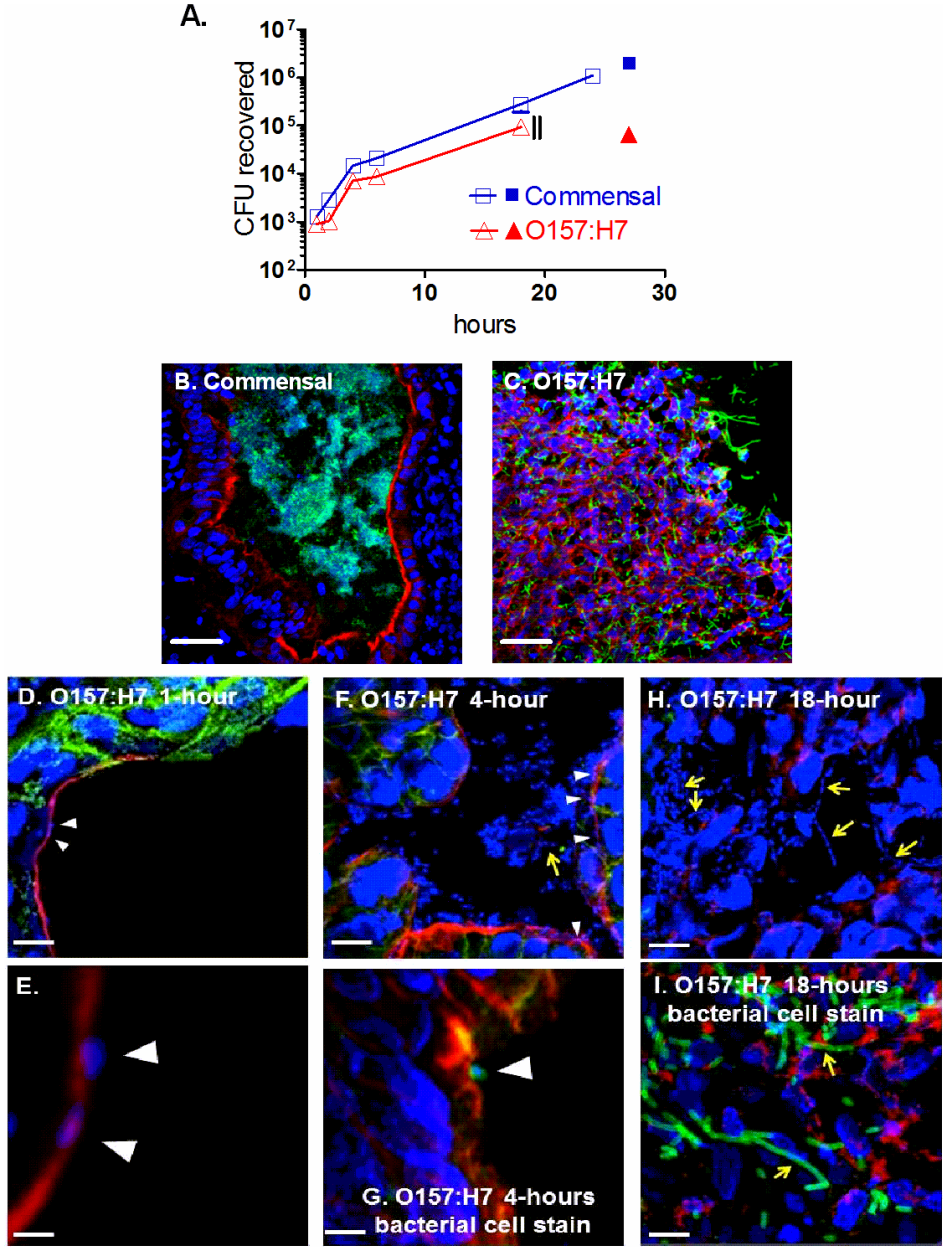
Bacterial growth in iHIOs. **A, Bacterial growth**. iHIOs were injected with 10^3^ commensal *E*. *coli* (SGUC183, squares), or pathogenic O157:H7 (PT29S, triangles). Open symbols, iHIOs were incubated with penicillin in the tissue culture medium to prevent bacterial growth outside of the lumen; mean CFU +/- standard deviation were determined at the indicated times from three different iHIOs for each strain. Closed symbols, in a separate experiment iHIOs were injected with 10^3^ commensal *E*. *coli* (SGUC183, squares), or pathogenic O157:H7 (PT29S, triangles) as above, but at 18 hours, the medium was replaced with medium lacking penicillin, and bacterial counts were assessed at 27 hours post inoculation. **B-C**, Commensal *E*. *coli* (B) replicates in the lumen without damaging the iHIO, while O157:H7 (C) damages the actin layer. Cryosections of iHIOs 18 hours after injection were stained for DNA (blue), bacteria (green, anti-*E*. *coli* for commensal, anti-O157 for O157:H7), and F-actin (red). Bar indicates 20 μm. **D-E**. Cryosection 1 hour after infection with O157:H7, stained for nuclear and bacterial DNA (DAPI, blue), E-cadherin (green), and F-actin (red). White arrowheads represent bacterial nucleoids co-localized with actin. (D), Bar indicates 10 μm. (E), Magnified image of D, bar indicates 2 μm. **F-G**. Cryosections 4 hours after infection with O157:H7. White arrowheads represent bacterial co-localization with actin. (F), stained for nuclear and bacterial DNA (DAPI, blue), E-cadherin (green), and F-actin (red), bar indicates 10 μm. (G), stained for nuclear and bacterial DNA (DAPI, blue), F-actin (red), and anti-O157 (green), bar indicates 5 μm. **H-I**. Cryosections 18 hours after infection with O157:H7. Yellow arrows indicate filamentous *E*. *coli*. (H), stained for nuclear and bacterial DNA (DAPI, blue), E-cadherin (green), and F-actin (red), bar indicates 10 μm. (I), stained for nuclear and bacterial DNA (DAPI, blue), F-actin (red), anti-O157 (green), bar indicates 10 μm. Representative images of experiments performed at least four times are shown.

To assess whether *E*. *coli* can replicate and persist in the iHIO lumen, approximately 10^3^ non-pathogenic commensal strain of *E*. *coli* (SGUC183) or clinical isolate (PT29S) of O157:H7, which only expressed Stx2a were microinjected into the iHIO lumen under conditions that prevented bacterial growth in the tissue culture medium. The iHIOs were able to support the growth of both *E*. *coli* strains with virtually identical growth rates, although the O157:H7 strain had a slightly longer lag phase (Fig 3A). Biphasic growth rates were observed. During the first 4 hours, both strains had a doubling time of about 30 minutes, similar to in vitro growth rates with aeration in nutrient rich medium. After about 4 hours, much slower doubling times of about 3 hours were observed, suggesting changes in the lumen environment, such as nutrient or oxygen depletion. At 24 hours about 10^6^ commensal bacteria were recovered, while after 72 hour over 10^7^ commensal bacteria were recovered (data not shown). Assuming iHIOs are hollow spheres about 0.1 cm in diameter (radius = 0.05 cm), the internal volume is equal to 4/3 πR^3^ (or 5.24 x 10^−4^ ml), for an estimated density of about 1.9 x 10^10^ bacteria per ml, within the range of bacterial density in the human ileum (about 10^8^ per ml) and colon (about 10^12^ per ml) [27–29].

At 24 hours, commensal *E*. *coli* but not pathogenic O157:H7 were recovered from the iHIOs. Furthermore, after 24 hours, organoids challenged with O157:H7 were fragile and often broke apart when removed from the extracellular matrix support. To determine if the inability to recover O157:H7 at 24 hours was due to loss of the epithelial barrier and subsequent exposure to the antibiotics from the tissue culture medium, at 18 hours the medium containing penicillin and streptomycin was replaced with antibiotic-free medium. The organoids were harvested at 27 hours post-infection (9 hours without antibiotics in the medium), and colony counts were assessed for both the tissue culture medium and the organoids. For the commensal, 2 x 10^6^ CFU were recovered from the organoid at 27 hours (Fig 3A, solid square), but no viable bacteria were recovered from the tissue culture medium, suggesting the commensal bacteria continued to replicate within the confines of the lumen. In contrast, nine hours after antibiotic removal, 6 x 10^4^ viable O157:H7 were recovered from the organoid (Fig 3A, solid triangle), and 3 x 10^5^ were recovered from the tissue culture medium. These results suggest that about 18 hours post-infection the O157:H7 destroy the luminal barrier, and are killed if antibiotics are present in the medium. However, if antibiotics are not present in the medium, they are capable of replicating in both compartments.

### Histologic characterization of iHIOs

Cryosections were examined to determine the effect of bacterial infection on iHIO morphology and the luminal epithelial layer. In sections taken at 18 hours, the epithelial layer of iHIOs injected with non-pathogenic SGUC183 was clearly defined by F-actin (red) and similar to PBS-injected organoids (Fig 1B), with numerous bacteria (green) within the lumen (Fig 3B). In contrast, the actin layer of iHIOs injected with O157:H7 (Fig 3C) was clearly disrupted, there was no evidence for a luminal compartment and filamentous bacteria (green) were present throughout the tissue.

Time-dependent damage to the iHIO was observed following injection of 10^3^ O157:H7 (Fig 3D–3I). At one hour (Fig 3D and 3E), while the lumen was clear, breaks in F-actin (red) were seen. At 4-hours post-infection (Fig 3F and 3G), disrupted F-actin and loss of E-cadherin expression was apparent. At 18 hours post infection (Fig 3H and 3I), the luminal border was gone, F-actin staining was sparse and randomly distributed, and the green E-cadherin staining was greatly diminished, with no obvious luminal structure.

iHIOs resemble the distal portion of the small intestine [12], the tissues that are favored for initial attachment of *E*. *coli* O157:H7 [14,30]. Adherence to human intestinal epithelium is a key determinant of pathogenicity. Strains possessing the locus of enterocyte effacement (LEE) display F-actin mediated intimate attachment to epithelial cells [31–34]. Individual bacteria could be seen in the expanded images. O157:H7 and other enteropathogenic *E*. *coli* display intimate attachment to intestinal epithelial cells mediated by the cytoskeletal protein F-actin [31]. Intimate contact can activate host antibacterial responses, such as production of reactive oxygen species (ROS), which in turn can induce expression of Stx through activation of SOS response in STEC [35,36]. At 1-hour post infection **(**Fig 3E, **white arrowheads)**, DNA the size of a bacterial nucleoid (blue staining) and F-actin (red) were co-localized as evidenced by the purple in expanded merged confocal image. Pedestal formation was not observed, although such structures are typically visualized by electron microscopy. At 4-hours post infection (Fig 3F) numerous bacteria were seen in the lumen, growing primarily as coccobacilli. Co-localization of DNA and F-actin was observed (Fig 3F, white arrowheads), and staining with the anti-*E*. *coli* antibody demonstrated that bacteria (green) were co-localized with the actin (Fig 3G, white arrowhead). At 18 hours post infection (Fig 3H), numerous filamentous DNA structures were seen. To verify that the filamentous structures were *E*. *coli* O157:H7, the organoids were stained with antibody to O157 LPS (Fig 3I). Numerous green coccobacilli as well as long green filaments were seen, demonstrating that the small, sub-nuclear DNA structures were *E*. *coli*.

### Production of reactive oxygen species (ROS)

**Fig 4:**
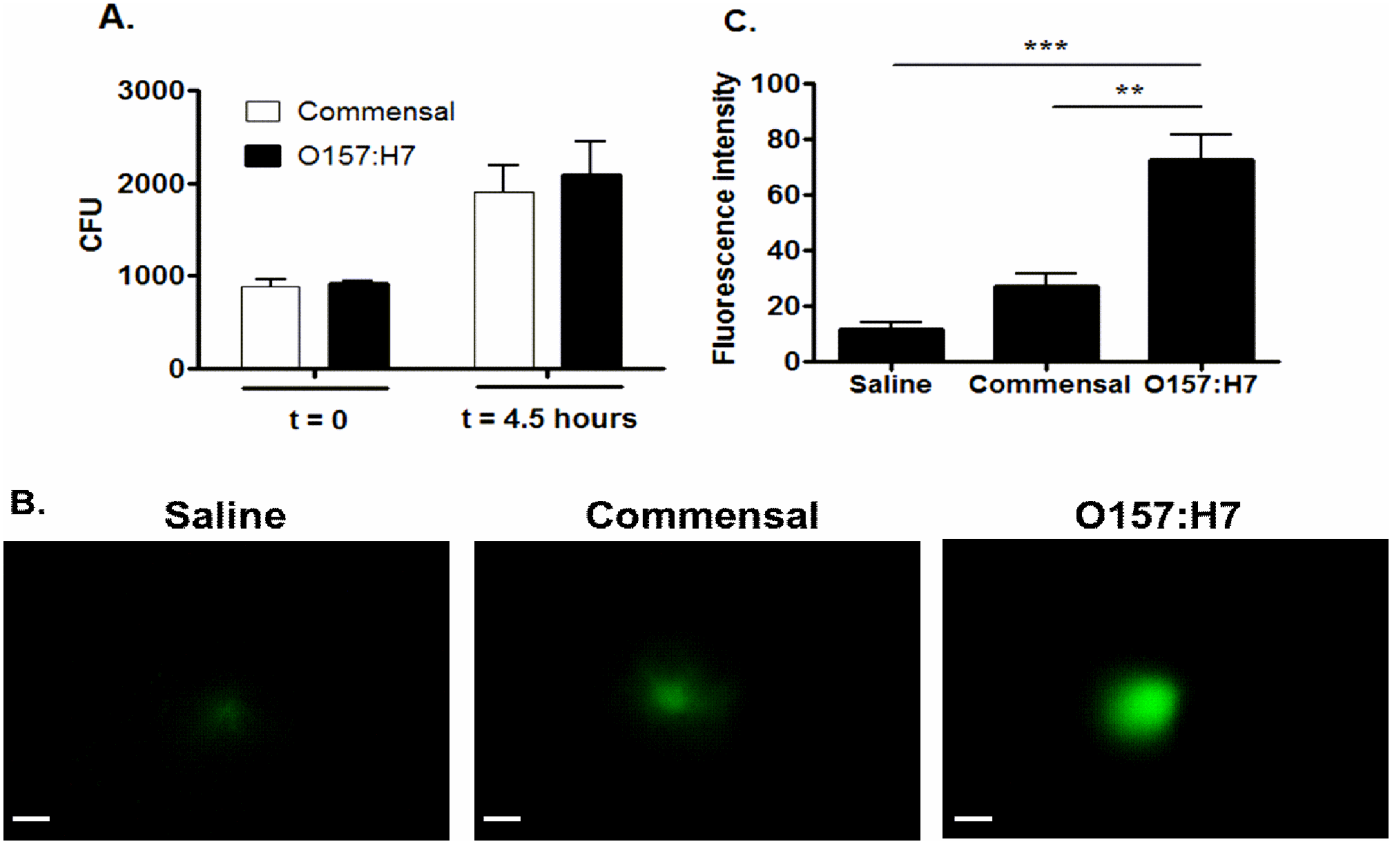
Infection with pathogenic O157:H7 upregulates ROS. (A) iHIOs were injected with saline and approximately 10^3^ commensal *E*. *coli* or pathogenic O157:H7. Mean CFU +/- standard error were determined at the indicated times from three different iHIOs for each strain. (B) ROS production was assessed 4 hours post-injection by fluorescence microscopy 30 minutes after addition of the oxidative stress detection reagent (Enzo). Bar indicates 100 μm. (C) Fluorescence intensity, pixels (mean and standard error of the mean) is plotted for three independent iHIOs. Statistical significance was assessed by using one-way ANOVA analysis of variance using Tukey’s multiple comparison posttest using GraphPad Prism 5.0. ** Very significant (*P* value 0.001 to 0.01), *** extremely significant (*P* value *p* < 0.001). Similar results were seen in an independent replicate experiment.

Filaments form when bacteria continue to replicate, but the daughter cells fail to separate, and occurs following exposure to DNA damaging agents, including ROS. The delay in septation is induced by the bacterial SOS system. It allows time for DNA damage repair, minimizing transfer of damaged chromosomes. ROS production was assessed in iHIOs were injected with saline, or 10^3^ commensal or 10^3^ pathogenic O157:H7. After 4 hours, bacterial recovery was similar for both strains (Fig 4A). Injection of O157:H7 resulted in significantly increased ROS compared to the saline control or injection with the commensal strain (Fig 4B and 4C).

### Stx production

**Fig 5:**
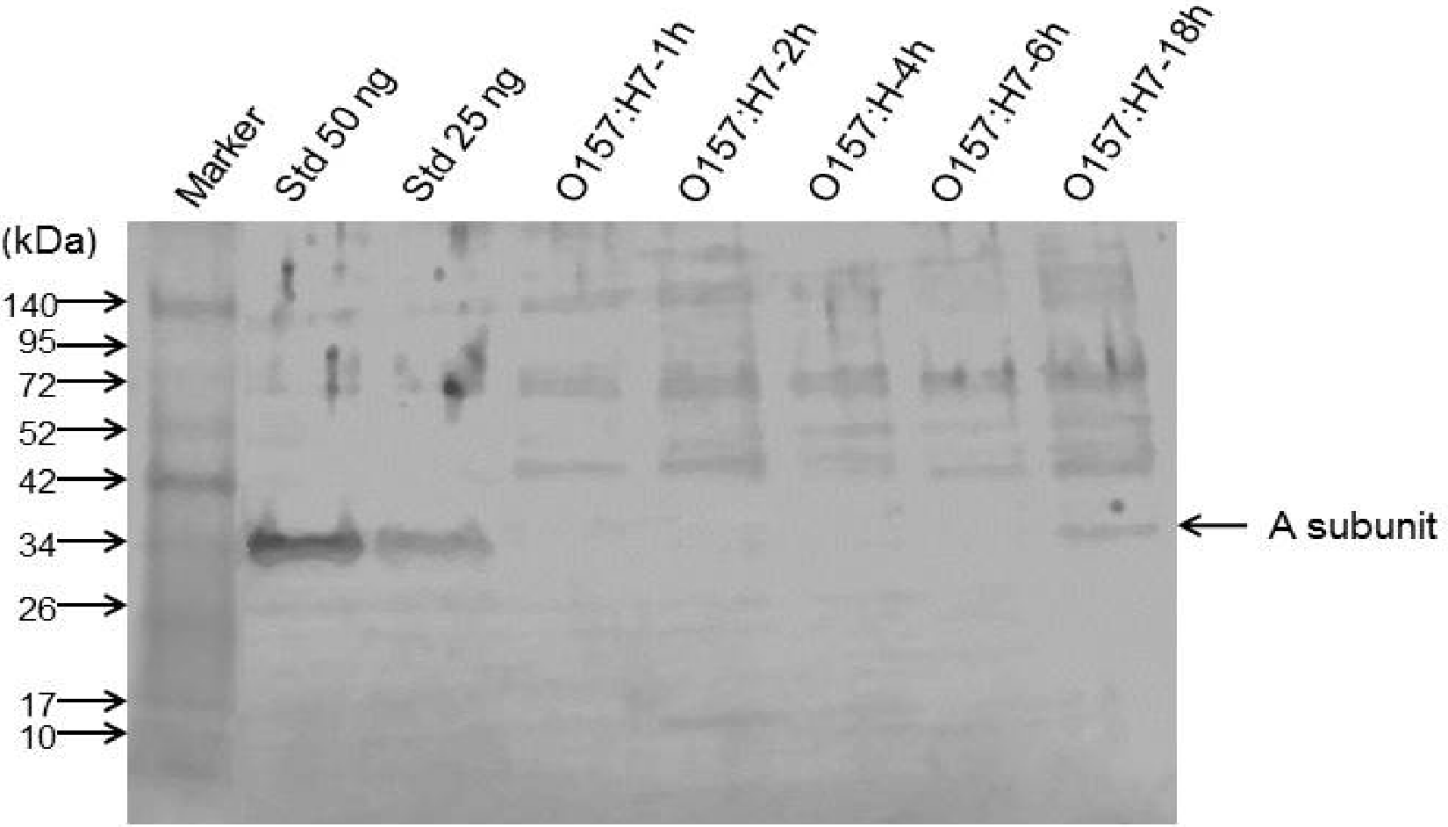
Western blot of Stx2a. Lysed iHIOs infected with *E*. *coli* O157:H7 were harvested at the indicated time points and mechanically lysed. The cellular material was collected by centrifugation. Protein in the pellet was solubilized, and immunoblotted using polyclonal antibody to Stx2a. As control, purified Stx2a was loaded at 50 and 25 ng. The Stx2a A-subunit (33 kDa), indicated by the arrow is seen at 18 hours post-infection.

While the SOS response is designed to protect chromosomal integrity, lysogenic bacteriophage use activation of the SOS response as a signal to initiate lytic replication and escape from a damaged host. Stx is phage-encoded, and activation of the SOS response initiates Stx expression [5]. Stx expression was observed in iHIOs infected with O157:H7. Stx2a was not detected by western blots at 1, 2, 4 and 6 hours post-infection (Fig 5). At 18-hours post infection, 4 ng and 10 ng Stx2a was detected in two separate experiments.

### Transcriptional profiling

#### Relative expression of linage specific genes

RNAseq was performed on iHIOs at 4 hours post-injection with PBS (control) or 10^3^ commensal *E*. *coli* or O157:H7. Expression of individual intestinal genes was examined (Table 1). Infection with the commensal and O157:H7 strain resulted in slight (approximately 2-fold), but significantly increased expression of epithelial junction proteins, including E-cadherin, ZO-2, and claudin 1. Both strains induced transcription of proteins that participate in gastrointestinal defenses, such as alkaline phosphatase, which is involved in detoxification of lipopolysaccharide (9 to 19-fold increase), the bacteriolytic enzyme lysozyme (6-fold increase), mucins involved in barrier function, including MUC2 (4 to 6 fold increase) and MUC13 (4-fold increase), and a structural component of gastric mucus, trefoil factor 2 (5-fold increase).

**Table 1:** Expression relative to PBS controls of select intestinal and lineage specific genes.

Symbol	Description	Cellular Distribution	O157:H7 4 hours Fold change (*P* value) ^a^	Commensal 4 hours Fold change (*P* value) ^a^
VIM	Vimentin	Mesenchyme	0.61 (0.02)	0.65 (0.2)
CDH1	cadherin 1, type 1, E-cadherin	Adherens Junction	2.3 (0.03)	2.4 (0.04)
CTNNB1	catenin beta 1	Adherens Junction	1.4 (0.2)	1.5 (0.3)
TJP1	tight junction protein 1 (ZO-1)	Tight Junction	0.89 (0.8)	0.91 (0.9)
TJP2	tight junction protein 2 (ZO-2)	Tight Junction	2.1 (0.01)	2.2 (0.02)
OCLN	Occludin	Tight Junction	1.7 (0.2)	2.2 (0.03)
CLDN1	claudin 1	Tight Junction	2.2 (0.02)	2.3 (0.04)
GJA1	gap junction protein, alpha 1	Gap Junction	1.8 (0.05)	1.8 (0.1)
VIL1	villin 1	Epithelial brush border	4.1 (0.01)	3.4 (0.12)
ALPI	alkaline phosphatase, intestinal	Enterocytes	19 (0.2)	9.2 (0.4)
LYZ	Lysozyme	Paneth cells	5.9 (0.0006)	5.9 (0.003)
MUC2	mucin 2	Goblet cells	6.1 (0.01)	4.4 (0.20)
MUC13	mucin 13, cell surface associated	Epithelial	4.3 (0.02)	4.1 (0.03)
CHGA	chromogranin A	Enteroendocrine cells	1.2 (0.95)	1.6 (0.9)
FUT2	fucosyltransferase 2	Mucosal layer	3.7 (0.03)	2.4 (0.4)
TFF1	trefoil factor 1	Mucosal layer	5.3 (0.002)	4.6 (0.02)
TFF2	trefoil factor 2	Mucosal layer	5.4 (0.03)	5.5 (0.06)
TFF3	trefoil factor 3 (intestinal)	Mucosal layer	3.0 (0.07)	2.2 (0.5)
A4GALT	Gb3 synthase (alpha 1,4-galactosyltransferase)		0.87 (1.0)	1.1 (1.0)
TLR4	toll-like receptor 4		0.91 (0.9)	0.95 (1.0)
IL1B	interleukin 1, beta		26 (2E-12)	13 (3E-06)
CXCL8	IL-8, chemokine (C-X-C motif) ligand 8		6.1 (0.04)	1.8 (0.8)
IL18	interleukin 18		6.7 (5E-05)	2.8 (0.3)
NLRC4	NLR family, CARD domain containing 4		0.0078 (0.03)	1.1 (1)
TNF	tumor necrosis factor		1.1 (1.0)	0.46 (0.9)
EGFR	epidermal growth factor receptor		0.62 (0.04)	0.53 (0.03)
MKI67	marker of proliferation Ki-67		0.52 (0.02)	0.70 (0.4)
NOX1	Tissue NADPH Oxidase		11 (0.17)	5.8 (0.40)

^a^ Adjusted for false discovery rate

#### Relative expression of immune response genes

The ability of infection to alter cellular signaling related to the innate immune defenses was also examined (Table 1). Bacterial infection constituted the first encounter of the sterile iHIOs with lipopolysaccharide (LPS); however, expression of the LPS receptor, TLR4 was not altered. Expression of IL-1β was highly upregulated by infection with either strain; however, infection with O157:H7, but not the commensal strain, resulted in significant upregulation of the inflammatory mediators, IL-8 and IL-18, and significant downregulation of NOD-like receptor, NLRC4.

### Transcriptional enrichment analysis

Setting significance at *P*<0.05 and using a 4-fold change compared to the PBS controls as the cutoff, infection with the commensal strain resulted in 317 differentially expressed genes (95 upregulated and 222 downregulated), while infection with the pathogenic O157:H7 strain resulted in 429 differentially expressed genes (160 upregulated and 269 downregulated). The most significantly (P< 3E-10) GO category uniquely upregulated by O157:H7 infection (S1A Fig) was “Chemokine-mediated signaling pathway” (GO:0070098), with upregulation of the genes indicated in S1A Fig, **box**. Other categories uniquely upregulated by O157:H7 included “regulation of response to wounding”, and the classical MAP kinase pathway, “positive regulation of ERK1 and ERK2 cascade”. Infection with either strain resulted in upregulation of the GO term, “digestive system process”. The most significant downregulated GO process category for both *E*. *coli* strains was the GO term, “Multicellular organismal process” (GO:0032501); with 82 down-regulated genes for the commensal strain and 88 for O157:H7.

**Fig 6:**
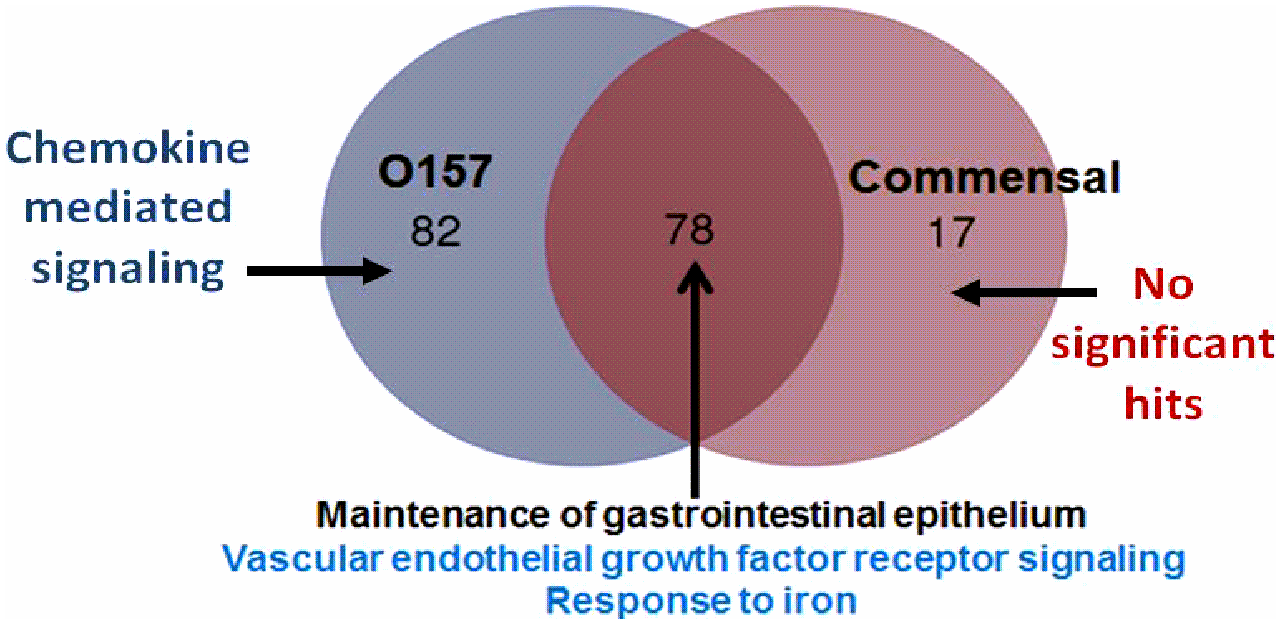
RNAseq—Venn diagram summary of upregulated genes. Venn diagram for the RNAseq analysis on iHIOs were prepared using an online tool from Bioinformatics & Evolutionary Genomics.

The upregulated GO terms were compared (Fig 6). Both commensal and O157:H7 infection up-regulated the GO terms “Response to iron” (GO:0010039) and “Regulation of vascular endothelial growth factor receptor signaling pathway” (GO:0030947), and “Maintenance of gastrointestinal epithelium” (GO:0030277). O157:H7 uniquely upregulated “Chemokine mediated signaling pathways” (GO:0070098).

### PMNs and infected iHIOs

**Fig 7:**
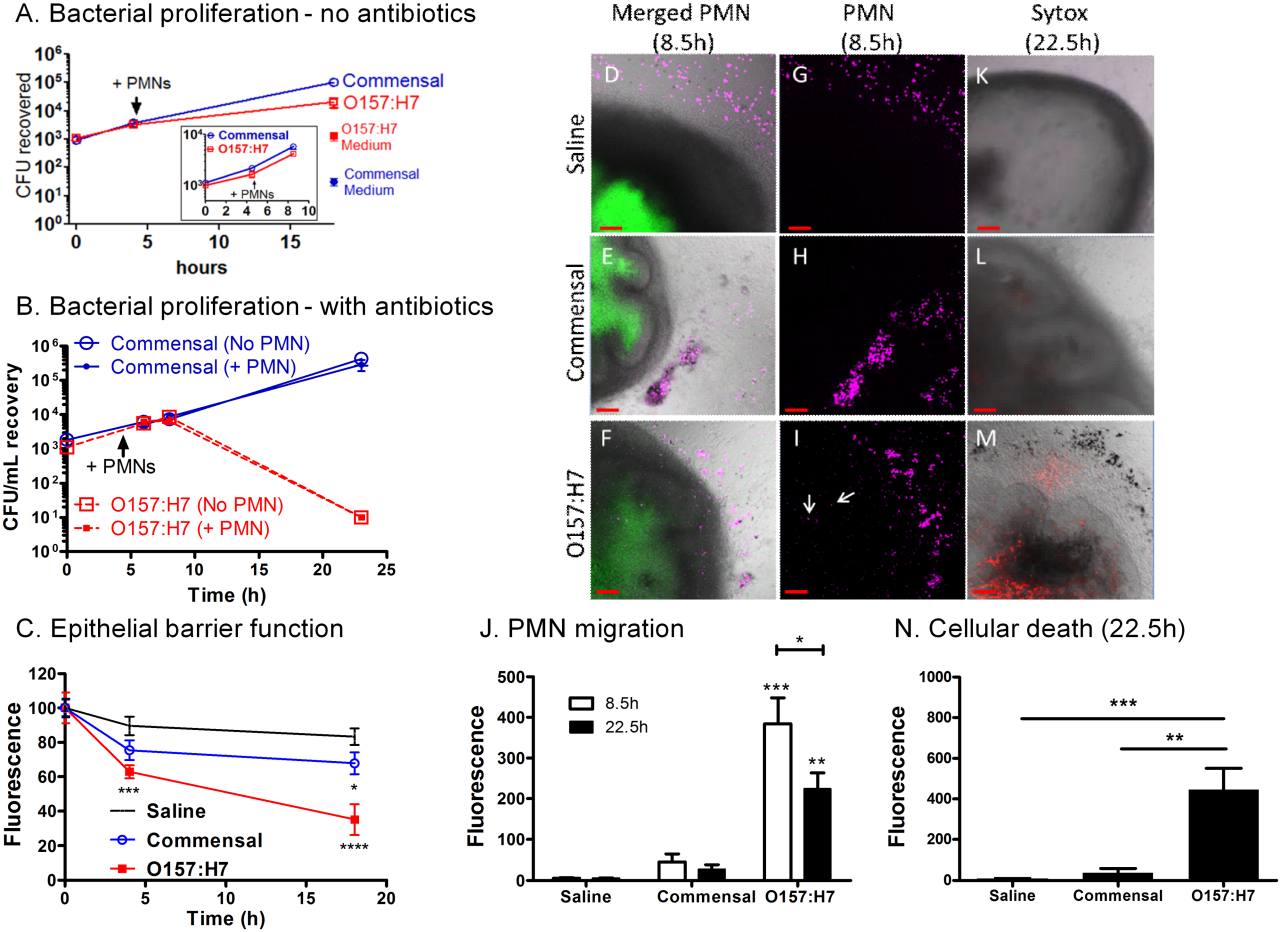
Influence of PMNs on bacteria and iHIOs. **(A-D)** iHIOs microinjected with saline or 10^3^ commensal bacteria or O157:H7 in the presence of the fluorescent dye FITC were incubated for approximately 4 hours to allow cytokine expression, and 5x10^5^ fluorescently labeled human PMNs were added. Incubations were performed without antibiotics in the tissue culture medium to assess total microbial growth, or with antibiotics in the tissue culture medium to assess maintenance of the luminal barrier. Each experiment was performed in triplicate. (A) Bacterial proliferation in the absence antibiotics. In two independent experiments, CFUs were determined before and after addition of PMNs (indicated by arrow). Insert, bacteria incubated with PMNs for 4.5 hours. Full graph, bacteria incubated with PMNs for 14.5 hours. Bacterial numbers within the iHIOs and media were assessed.(B) Bacterial proliferation in the presence of antibiotics. CFUs were determined at the indicated times before and after addition of PMNs (indicated by arrow). Failure to recover O157:H7 at 23 hours is due to antibiotics in the lumen following loss of the epithelial barrier. (C) Epithelial barrier function, assessed by retention of FITC fluorescence, for experiment Part A, full graph. Significance was calculated by two-way ANOVA: *, P < 0.05; ***, P < 0.001; ****, P < 0.0001. (D-J), **PMN migration into iHIOs**. **(D-F)**, merged bright field images of iHIOs, green FITC fluorescence (lumen), and violet PMNs, for iHIOs (from **Fig 7A, insert**) injected with saline, commensal, or pathogenic O157:H7, respectively, 8.5 hours after injection (4.5 hours after addition of PMNs). (G-I), violet fluorescent images, corresponding to D-F. (J), quantification of PMNs (violet fluorescence) within the iHIOs. Statistical analysis determined by 1-way AVOVA, Tukey’s multiple comparison posttest; ***, extremely significant, P < 0.001, comparing O157:H7 to saline and commensal (8.5 hours); **, very significant, P 0.001 to 0.01, comparing O157:H7 to saline and commensal (22.5 hours) and *, significant, P < 0.05 comparing O157:H7 (8.5 hours) to O157:H7 (22.5 hours). (K-M), merged bright field images of iHIOs (from **Fig 7A, full graph**) and sytox-orange dead-cell stain, 22.5 hours after injection (18.5 hours after addition of PMNs). (N), quantification of dead cells (red fluorescence due to sytox-orange staining). Statistical analysis determined by 1-way AVOVA, Tukey’s multiple comparison posttest: ***, extremely significant, P < 0.001: **, very significant, P 0.001 to 0.01. Red bar on micrographs indicates 100 μM.

The inflammatory mediator, IL-8, is associated with neutrophil recruitment, alternatively breach of the intestinal barrier could promote recruitment by the presence of pathogen-associated molecular patterns, such as LPS. We assessed whether pathogenic O157:H7 promoted neutrophil recruitment. iHIOs were injected with saline, or 10^3^ commensal or O157:H7, in the presence of the fluorescent dye FITC to label the lumen, and incubated for four hours to allow for chemokine expression. Human PMNs (polymorphonuclear leukocytes), a population comprise primarily of neutrophils, were labeled with fluorescent cell-tracker dye, and 5 x 10^4^ were added to the medium. Initial studies were done in the absence of antibiotics to allow for assessment of the potential of PMNs to reduce bacterial numbers (Fig 7A).

Bacterial recovery from the iHIOs was similar at 4 hours before addition of the PMNs. Both strains grew within the organoid after addition of the PMNs, and at 18 hours bacterial recovery from the iHIO in the presence of PMNs (Fig 7A, **full graph**) was not statistically different from growth in the absence of PMNs (Fig 7A). The culture medium was also sampled. About 1300 O157:H7 were recovered from the media, but only 32 commensal bacteria were recovered from the media, suggesting O157:H7 may have breached the epithelial barrier.

As shown in Fig 3A, antibiotics can access and kill the bacteria if the epithelial barrier is breached. The influence of antibiotics in the tissue culture medium on bacterial recovery was assessed in the presence and absence of PMNs (Fig 7B). At 6 and 8 hours, bacterial recovery was similar in the presence or absence of PMNs. However, at 23 hours post-infection, the commensal strain was recovered whether or not PMNs were present, but no viable O157:H7 were recovered. This is consistent with O157:H7 induced loss of the intestinal barrier, and further suggests that the presence of PMNs cannot prevent the epithelial damage. Epithelial barrier function was further evaluated by quantifying fluorescence of FITC injected into the lumen (Fig 7C and 7D–7F). Fluorescence in the iHIOs injected with O157:H7 was significantly reduced compared to the saline controls at 4 and 18 hours (Fig 7C), while significantly less fluorescence was recovered from the iHIOs injected with the commensal strain at 18 hours.

#### Recruitment of PMNs

Recruitment of PMNs was monitored by microscopy (Fig 7D–7I). In the merged bright field and fluorescent images, the dark iHIO with a green, FITC-labeled lumen can be seen. PMNs (violet) were seen at the periphery of all iHIOs (Fig 7D–7F and 7G–7I). For injection with saline or commensal, violet cells were primarily localized to the periphery of the iHIO. In contrast, for injection with O157:H7, violet cells were seen at the periphery, as well as within the iHIO and in some cases co-localize with the green stain that defines the lumen (Fig 7I, **white arrows**). The violet signal within region corresponding to the body of the iHIO was quantified. Significantly more fluorescent signal was detected in the iHIOs infected with *E*. *coli* O157:H7 than the saline or commensal-infected iHIOs at both 8.5 and 22.5 hours (Fig 7J). Cellular death was assessed with Sytox Orange, a membrane impermeant dye that stains cellular nucleic acids if the membrane has been compromised (Fig 7K–7M). Significantly more fluorescent signal was detected in iHIOs infected with *E*. *coli* O157:H7 compared to saline or commensal-infected iHIOs (Fig 7N).

**Fig 8:**
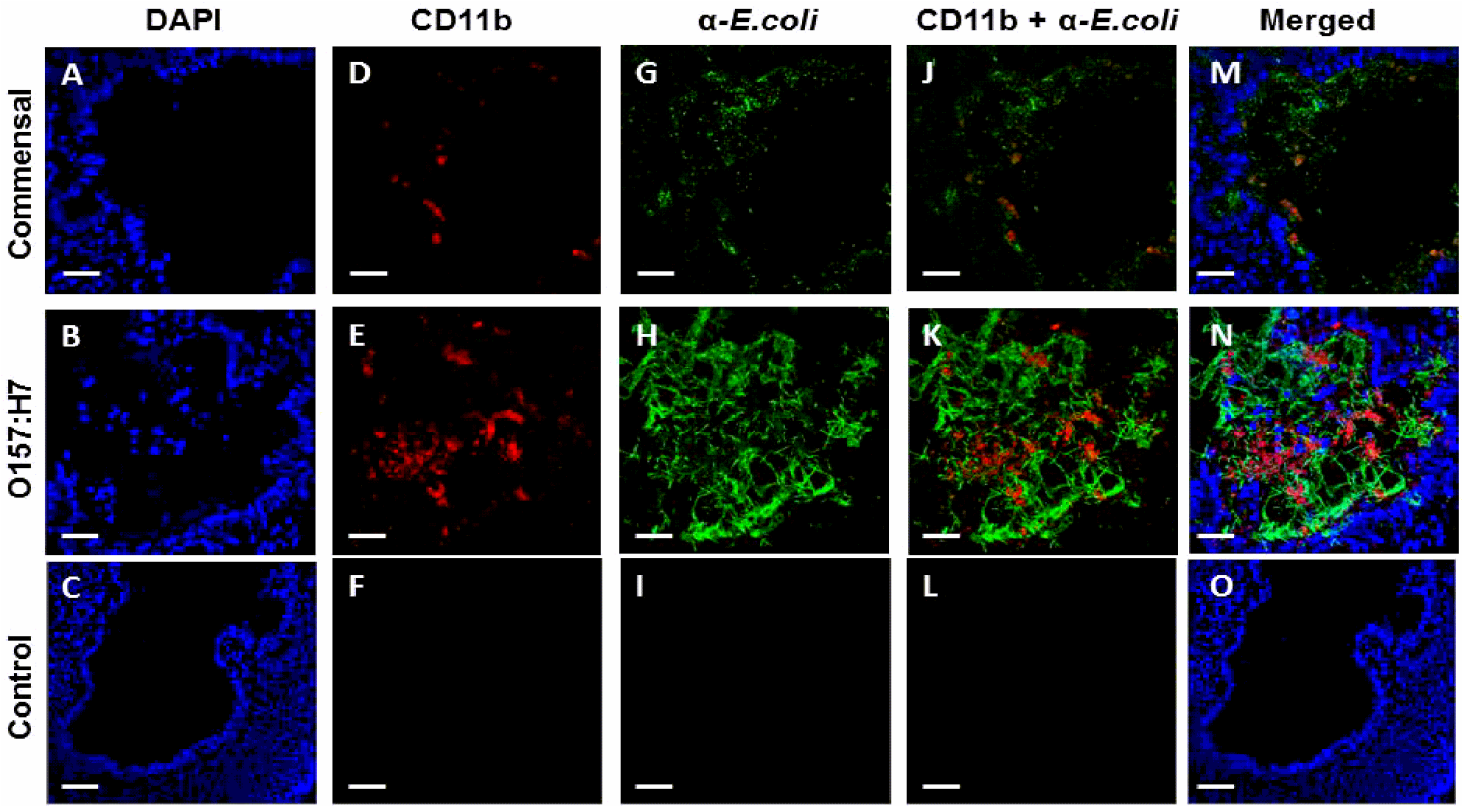
Recruitment of PMNs to the site of infection. Confocal images of cryosections of iHIOs, stained for nuclear DNA (blue), CD11b (red) and antibody to *E*. *coli* (α*-E*.*coli*, green) injected with commensal, pathogenic O157:H7 or saline, respectively, 22.5 hours after injection (18.5 hours after addition of PMNs). (A-C), DAPI nuclear staining, (D-F) human neutrophil marker CD11b staining (G-I) anti *E*.*coli* antibody for commensal and anti O157:H7 antibody against O157:H7 (J-L), merged CD11b and α*-E*.*coli* stains only and (M-O), merged images of cryosections of commensal, O157:H7 and saline respectively. Note the filamentous growth of O157:H7. Bar indicates 100 μM.

Luminal presence of the phagocyte marker, CD11b, was also monitored in cryosections (Fig 8). No CD11b signal was seen in control iHIOs injected with saline (Fig 8O). Some PMNs were in the lumen of the commensal infected iHIOs, as evidenced by red fluorescence (Fig 8M) and more were observed in the iHIOs infected with O157:H7 (Fig 7N). Less bacterial staining was observed for the commensal *E*. *coli* compared to *E*. *coli* O157:H7, likely due to the use of different bacterial antibodies, since Fig 7A demonstrated recovery of the two strains was similar.

## Conclusions

Commensal *E*. *coli* grew to high numbers in the previously sterile iHIO lumen without causing damage, demonstrating that like the neonatal intestine, the innate defenses of iHIOs are sufficient to contain non-pathogenic bacteria [37]. Tolerance of commensal bacteria is also seen in wild type mice, as well as severely immunodepleted NOD scid gamma (NSG) mice, lacking mature T cells, B cells, and natural killer (NK) cells. In contrast, growth of pathogenic O157:H7 resulted in loss of epithelial barrier function. Thus some property or properties expressed by pathogenic O157:H7, but not commensal *E*. *coli*, is responsible for the rapid loss of epithelial barrier function. Both strains have been sequenced, and a likely candidate is the O157:H7 LEE pathogenicity island, which is known to alter the integrity of the actin cytoskeleton, a cellular component necessary to maintain epithelial cell contact. A second candidate is Shiga toxin, which is known to kill cells. Whether either, both, or neither traits mediate the phenotype observed with O157:H7 infection could be resolved by experiments with defined mutants in O157:H7.

Pathogenic O157:H7 activated innate defenses, including ROS production (Fig 4) and several inflammatory immune responses (S1A Fig, Table 1). The different bacterial morphologies are consistent with differential activation of the host defenses [35]. The commensal strain grew normally as cocco-bacilli, while O157:H7 displayed filamentous growth (Figs 3 and 8).

In human disease, elevated neutrophil counts have been associated with development of HUS and fatal outcome [38,39]. IL-8 induces neutrophil-chemotaxis, and was upregulated by O157:H7. PMNs accumulated at the iHIO margins, migrated through the tissue and localized within the lumen. However, recruitment of PMNs did not prevent loss of epithelial barrier function (Fig 7B and 7C) or reduce the O157:H7 numbers (Fig 7A). This could be since long filamentous chains, as seen for O157:H7, can protect bacteria from phagocytosis [40,41]. Recruitment and activation of PMNs could contribute to tissue damage without helping to resolve the infection.

Lack of experimental models has hampered investigation of human-restricted pathogens such as *E*. *coli* O157:H7. Our studies comparing infection of commensal to pathogenic *E*. *coli* demonstrate iHIOs represent a valuable model to study human-restricted enteric pathogens.

## Supporting information

S1 Fig A, Highly significant GO PROCESS pathways upregulated by (A) O157:H7, PT29S and (B). Commensal SGUC183.
(DOCX)

S1 Table Antibodies, stains, and reagents used in this study. (DOCX)

S2 Table RNAseq 4 hours post infection with commensal *E*. *coli* versus PBS (samples in triplicate). (XLSX)

S3 Table RNAseq 4 hours post infection with O157:H7 versus PBS (samples in triplicate). (XLSX)
