# Supplementary material for: Intestinal organoids model human responses to infection by commensal and Shiga toxin producing Escherichia coli
Source: PLoS One. 2017 Jun 14;12(6):e0178966. doi: 10.1371/journal.pone.0178966 (PMC5470682; doi:10.1371/journal.pone.0178966)

**
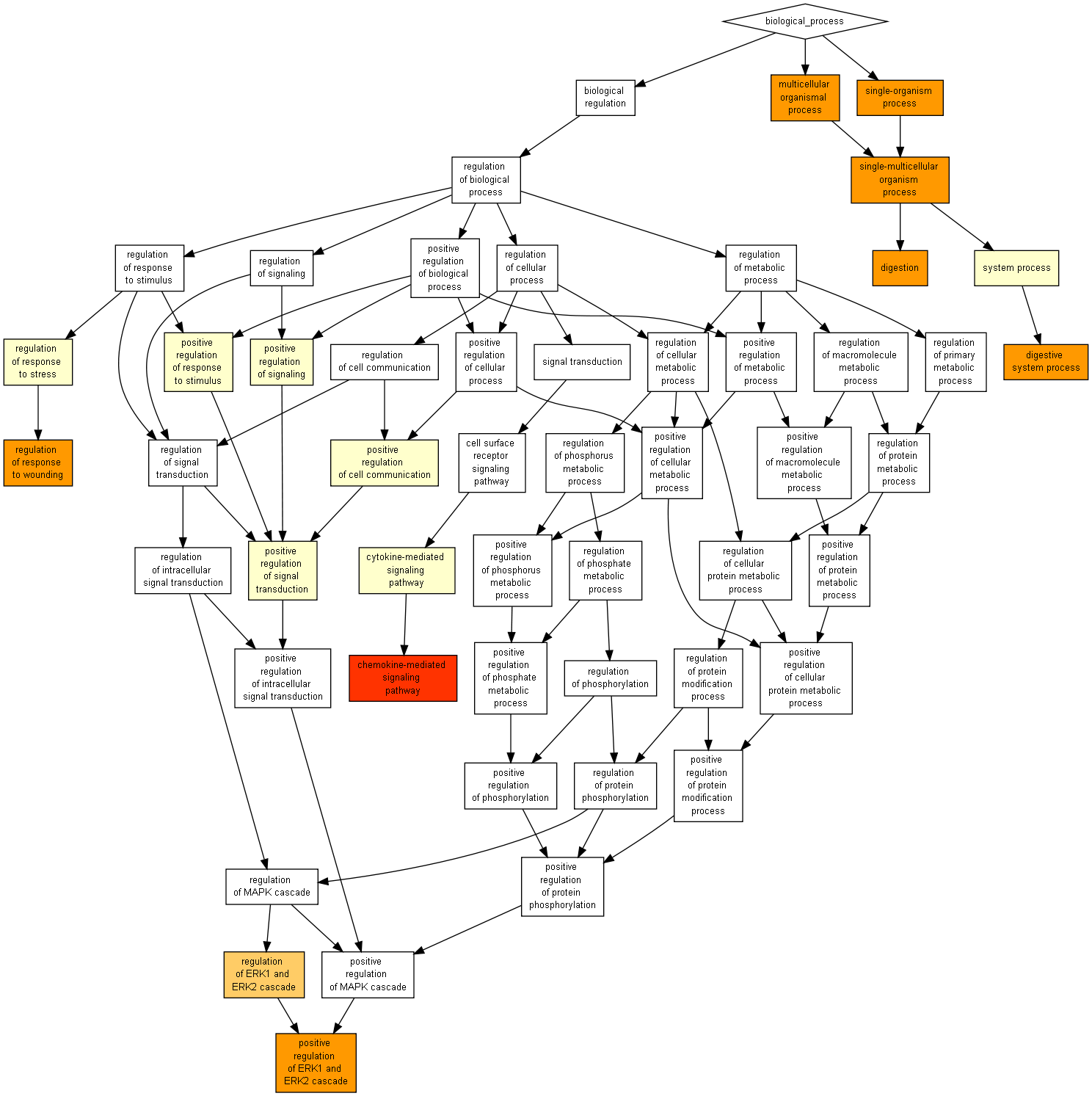
Figure S1 A. Highly significant GO PROCESS pathways upregulated by O157:H7,** PT29S (nested term with cutoff of *P*<10^-7^ displayed), with significantly upregulated chemokine pathway genes listed next to box.


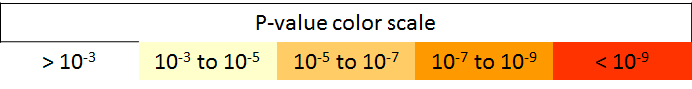


P value color scale

**Chemokine-mediated Signaling Pathway Upregulation of:**

**CCL3L3**

**CXCL5**

**CCL15**

**IL8**

**CCL4**

**CXCL10**

**CCL3L1**

**CCL3**

**trefoil factor 2**

**Figure S1 B. Highly significant GO PROCESS pathways upregulated by commensal SGUC183** (nested with cutoff of *P*<10^-6^ displayed).


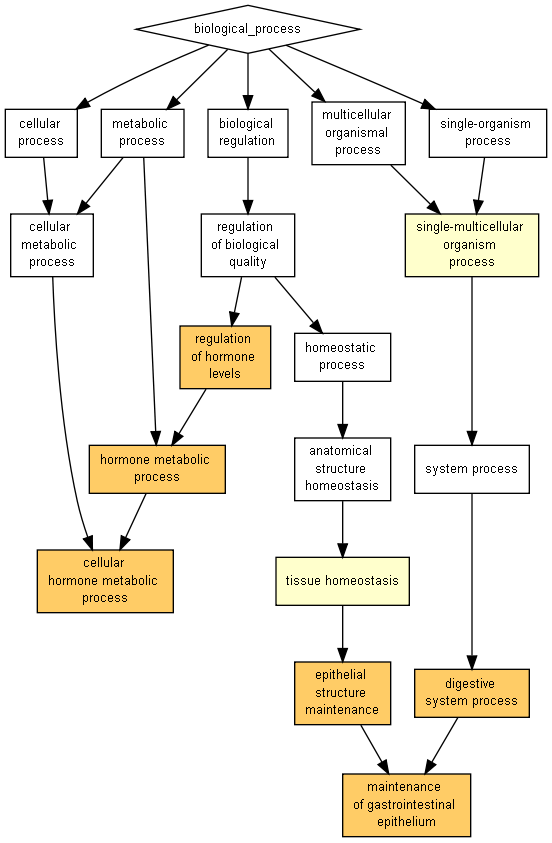

Supplement: S1 Fig — Commensal SGUC183. (DOCX) [file pone.0178966.s001.docx]
