# Supplementary material for: Intestinal organoids model human responses to infection by commensal and Shiga toxin producing Escherichia coli
Source: PLoS One. 2017 Jun 14;12(6):e0178966. doi: 10.1371/journal.pone.0178966 (PMC5470682; doi:10.1371/journal.pone.0178966)
Supplement: S1 Table — (DOCX) [file pone.0178966.s002.docx]

**S1 Table. Antibodies, stains, and reagents used in this study**

| **Cellular marker** | **Specificity** | **Cellular**  **Localization** | **Dye / Antibody (Monoclonal/**  **Polyclonal)** | **Source** |
| --- | --- | --- | --- | --- |
| DNA | All cells | Nucleus / nucleoid | Hoechst 33342 | Invitrogen  (H-3570) |
| DNA | All cells | Nucleus / bacterial nucleoid | 4',6'-diamidino-2-phenylindole (DAPI), | Invitrogen (D3571) |
| Fluorescein isothiocyanate |  |  | Fluorescent dye | Molecular Probes  (F-1906) |
| F-actin | Polymerized actin | Cytoskeleton | Texas-red phalloidin | Molecular Probes (T7471) |
| Anti-E-cadherin | Epithelial cells | Adherens junction | HECD-1, mouse monoclonal | abcam  (ab1416) |
| Purified LPS | Gram negative bacteria | Outer membrane |  | InvivoGen LPS-EB ultrapure (tlrl-3pelps) |
| Purified Stx2a |  |  |  | BEI Resources  (NR-4478) |
| Anti-Shiga toxin | Stx2 A-and B-subunits |  | Rabbit anti-recombinant Stx1 A and B subunits | Pellino, et. al. J Bacteriol.2016;198:1621-1630. Doi:10.1128/JB.0091  8-15 |
| Anti-O157 | *E. coli* serotype | LPS | 3011, mouse monoclonal | abcam  (ab20976) |
| Anti-*E.coli* | “O” and “K” antigenic serotypes | Cell surface | Rabbit polyclonal | Virostat (1001) |
| Anti-CD11b | Macrophage marker | Leukocytes | Rabbit polyclonal | Abcam (ab82039) |
| Secondary Anti-Mouse | IgG | Gamma Immunoglobins (H + L) | Goat polyclonal | Life Technologies (A11001) |
| Secondary  Anti-Rabbit | IgG | Gamma Immunoglobins (H + L) | Goat polyclonal | Life Technologies (A11012) |
| Secondary Anti-Rabbit | IgG | Gamma Immunoglobins (H + L) | Goat anti rabbit  Fluorescent antibody | *LI-COR*  (926-32211) |
| CellTracker^TM^ Violet BMQC dye | Neutrophils | Cellular | Dye | Life Technologies  (C10094) |
| ROS detection reagent |  |  | Indicator for presence of reactive oxygen species | Enzo Life Sciences  (ENZ-51011) |
